# Gut Microbiota, the Potential Biological Medicine for Prevention, Intervention and Drug Sensitization to Fight Diseases

**DOI:** 10.3390/nu14204220

**Published:** 2022-10-11

**Authors:** Haijie Wu, Xiaoguang Chen, Sen Zhang, Jiaxin Li

**Affiliations:** 1 State Key Laboratory of Bioactive Substances and Functions of Natural Medicines, Institute of Materia Medica, Chinese Academy of Medical Sciences & Peking Union Medical College, Beijing 100050, China; 2 Department of Gastrointestinal Surgery, The Sixth Affiliated Hospital of Wenzhou Medical University & Lishui City People’s Hospital, Lishui 323000, China

**Keywords:** gut microbiota, *Akkermansia*, *Bifidobacterium*, *Lactobacillus*, *Parabacteroides*, cancer, neurodegenerative diseases, type II diabetes, kidney diseases, autism spectrum disorder

## Abstract

As the largest “immune organ” of human beings, the gut microbiota is symbiotic and mutually beneficial with the human host, playing multiple physiological functions. Studies have long shown that dysbiosis of gut microbiota is associated with almost all human diseases, mainly including type II diabetes, cancers, neurodegenerative diseases, autism spectrum disorder, and kidney diseases. As a novel and potential biological medicine for disease prevention, intervention and drug sensitization, the gut microbiota has attracted more and more attention recently. Although the gut microbiota is a comprehensive microbial community, several star bacteria have emerged as possible tools to fight against various diseases. This review aims to elucidate the relevance of gut microbiota dysbiosis with disease occurrence and progression, and mainly summarizes four well-known genera with therapeutic and sensitizing potential, *Akkermansia*, *Bifidobacterium*, *Lactobacillus* and *Parabacteroides*, thoroughly elucidate their potential value as biological drugs to treat diverse disease.

## 1. Gut Microbiota

The human body contains more than 10^14^ microorganisms, of which about 70% of the microbial symbiotic community colonizes the gastrointestinal tract, namely the gut microbiota, which is a complex microbial community and makes the gastrointestinal tract the largest interface between the human body and the external environment [1,2]. The total number of genes of microorganisms contained in the gut microbiota is about 100 times that of the human genome, as well as the fact that they also endow many functions that humans do not have [3]. The co-evolution of the host and the microbe over millions of years promotes a symbiotic relationship, in which the microbiota contributes to many physiological processes of the host, and the host provides the microbe with a nutrient-rich and habitable environment [4]. The gut microbiota is known to provide many benefits to our body, including pathogen protection, nutrient absorption and synthesis, metabolism and immune response, etc. [5]. In recent years, studies have shown that gut microbiota dysbiosis is also linked to a variety of diseases, including tumors, neurodegenerative diseases, type 2 diabetes, kidney disease, autism, and so on [6,7,8,9].

## 2. Classification and Function of Gut Microbiota

The gut microbiota is dominated by bacteria, but also includes viruses, archaea, fungi and other eukaryotes [10]. Bacteria dominate the gut microbiota, with more than 1000 species, more than 90% of which belong to *Bacteroidetes* and *Firmicutes*, and the rest of the bacteria are included in four main phyla (*Actinobacteria*, *Fusobacterium*, *Proteobacteria*, *Verrucomicrobia*) and other minor phyla [11]. The colonization of humorous gut microbiota has also brought a series of benefits and played an important role in maintaining the normal physiological functions of the body.

### 2.1. Pathogen Protection

The protective effect of gut microbiota on pathogens in the gastrointestinal tract can be roughly divided into two aspects: physical and biological. Physically, gut microbiota can play a protective role by reducing the exposure of pathogen epitopes via colonizing intestinal epithelial cells. Germ-free mice are more susceptible to intestinal damage than normal mice, and it can be reversed by microbial colonization, suggesting that commensal colonization reduces pathogen epitope exposure and susceptibility to intestinal damage [12]. Biologically, gut microbiota can play a protective role by producing bacteriocins, indoles, extracellular vesicles, and short-chain fatty acids (SCFAs) [13]. Bacteriocins are polypeptides produced by certain bacteria that can kill or inhibit the growth of pathogens [14]. *Bacillus thuringiensis* DPC 6431, isolated from human feces, inhibits the growth of the *Clostridium difficile* by producing the bacteriocin Thuricin CD [15]. In addition, other important biological functions are summarized as follows.

### 2.2. Synthesis and Absorption of Nutrients

The gut microbiota plays a pivotal role in the synthesis of essential vitamins that the body cannot synthesize. Abundant research has shown that the gut microbiota can synthesize a variety of vitamins, especially vitamin K and B, including biotin (B7), cobalamin (B12), folic acid (B9), niacin (B3), pantothenic acid (B5), pyridoxine (B6), riboflavin (B2) and thiamine [16]. María P. Taranto et al., reversely demonstrated that *Lactobacillus reuteri* CRL1098 can produce vitamin B12 using a sugar-glycerol co-fermentation reaction in a medium without vitamin B12 [17]. Some scholars have shown that certain *Bifidobacteria* and *Lactobacilli* can synthesize vitamins (such as folic acid), and intake of these bacterial preparations can increase the content of folic acid in the intestine and plasma [18].

### 2.3. Metabolism

The gut microbiota is involved in various metabolic processes in our body, including energy, glucose and lipid metabolism [19]. The gut microbiota contains various carbohydrate-degrading enzymes, such as glycoside hydrolase (alpha amylase, type 1 pullulanase), polysaccharide lyase (inulin lyase, inulinase), carbohydrate esterase (poly Galacturonidase, acetylesterase, pectin lyase, pectin methylesterase), can degrade indigestible carbohydrates so that they can be used by the human body and the rest of the microbiota [20].

### 2.4. Immune System

The role of gut microbiota on host immunity has been extensively studied in germ-free animals. Germ-free mice have numerous immunodeficiencies, including low expression of antimicrobial peptides in the epithelium, reduced T lymphocyte numbers and activation, reduced plasma cell numbers, and impaired IgA production [21]. June L. Round et al. found that Foxp3+Treg cells were reduced in germ-free mice, while mice colonized with intestinal bacteria *Bacteroides fragilis* could proliferate and activate these cells [22]. SCFAs produced by gut microbiota metabolism can up-regulate anti-inflammatory and down-regulate pro-inflammatory cytokines through different mechanisms, resulting in a comprehensive anti-inflammatory effect [23].

### 2.5. Drug Biotransformation

The diverse gut microbiota naturally contains a variety of metabolic enzymes, so the impact on drugs is obvious. The gut microbiota and its enzymatic products and subsequent products, such as SCFAs and bile acids, play an important role in the biotransformation of drugs by directly or indirectly affecting their absorption, toxicity, metabolism, and bioavailability [24,25]. It is widely known that gut microbiota (such as *Bifidobacterium* H1) can exert metabolic activity by converting polar ginsenosides to non-polar ginsenosides through enzymes such as β-glucosidase [26,27]. Enterohepatic circulation of mycophenolate mofetil (MMF) requires gut microbiota to convert stable phenolic glucuronide (MPAG) without pharmacological activity into active mycophenolic acid (MPA) via β-glucuronidase (GUS) [28]. 5-Aminosalicylic acid (5-ASA) is rapidly absorbed orally and cannot play a role in the intestinal mucosa of inflammatory bowel disease (IBD) patients, so its prodrug, Olsalazine, was developed. Olsalazine is composed of two 5-ASA molecules linked by a diazonium bond, which is poorly absorbed in the upper gastrointestinal tract, but in the large intestine, the diazonium bond is cleaved by anaerobic and aerobic bacteria to generate 5-ASA, which is used to exert its medicinal effect [29]. Although there are many examples of drug toxicity and bioavailability reduction caused by gut microbiota, there are also many examples of drug efficacy through the design of prodrugs. More in-depth research is still needed to serve the clinic and making full use of the intestinal flora for the biotransformation of drugs can also benefit humans.

## 3. The Relationship between Gut Microbiota and Disease

**Figure 1:**
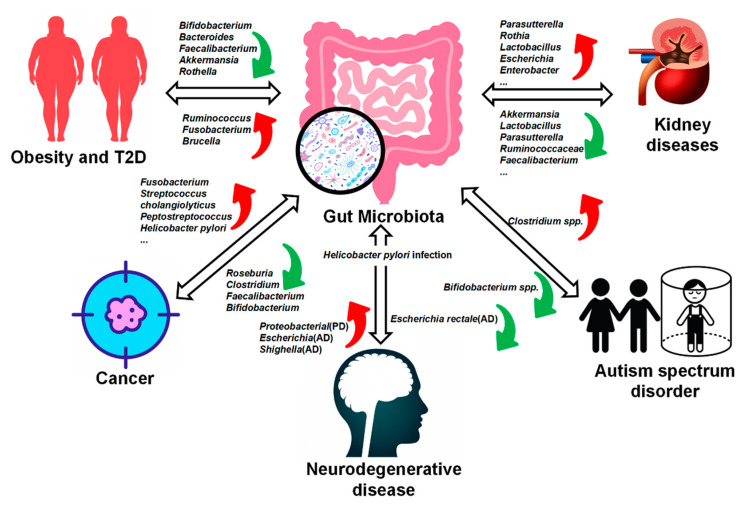
The relationship between gut microbiota and disease. Many diseases alter the composition of the gut microbiota, which also plays a key role in disease progression. Gut microbiota changes in patients with obesity, Type II Diabetes(T2D), cancer, psychiatric disorders, autism spectrum disorder, and so on. Metabolites of gut microbiota have also been implicated in some disease processes, such as cardiovascular disease. Gut microbiota offers new direction for disease treatment. Note: Alzheimer’s disease, AD; Parkinson’s disease (PD). The meaning of a symbol in the table: red arrow, up-regulated; green arrow, down-regulated.

In recent years, more and more scientists have realized the importance of gut microbiota to the body. The study of intestinal flora has been one of the hotspots for the last decade. With the continuous development of 16S rRNA technology, more and more studies have shown that the imbalance of gut microbiota is inseparable from many diseases. Below, we will focus on the following key diseases for a detailed review (Figure 1), whose supplements are shown in Table 1.

### 3.1. Obesity and Type II Diabetes (T2D)

Obesity is one of the most prevalent problems in the world, which is caused by excessive accumulation of fat, and there are various metabolic abnormalities, of which insulin resistance can also easily lead to diabetes. Routine feeding of germ-free (GF) animals versus normal animals found that although normal mice ate less than GF mice, they had 42% more total fat and 47% more gonadal fat than GF mice, indicating obesity is related to gut microbiota [30]. Compared with normal individuals, obese individuals had reduced gut bacterial diversity, with some bacteria taxa elevated, such as *Firmicutes*, *Proteobacteria*, *Fusobacterium*, *Lactobacillus*, and *Firmicutes*/*Bacteroidetes* ratios, while others are reduced, such as *Bacteroidetes*, *Faecalibacterium palau*, *Akkermansia*, *Methanobacter smithii*, and *Bifidobacterium* [31]. Recent research on obesity-related probiotics is relatively sufficient. *Hafnia alvei* HA4597 and *Bifidobacterium animalis* subsp. *lactis* 420 (B420) have shown good effects in animals and clinical practice with good safety [32,33,34]. In addition, *Akkermansia*, a new generation of probiotics, will be detailed later.

The prevailing view is that T2D is one of the attributes of obesity, and it is estimated that more than 80% of patients with T2D are overweight [35]. In the reported studies, the genera *Ruminococcus*, *Fusobacterium*, and *Brucella* were positively associated with T2D, while *Bifidobacterium*, *Bacteroides*, *Faecalibacterium*, *Akkermansia*, and *Rothella* were negatively associated with it [36]. An elevated proportion of Gram-negative bacteria rich in lipopolysaccharide (LPS), increased cellular permeability, decreased beneficial SCFA-producing bacteria, and diminished gut protection, resulting in low-grade systemic inflammation considered one of the immune mechanisms of T2D [37].

### 3.2. Cancer

It has been discovered that the gut microbiota is closely related to the occurrence and development of a variety of cancer types in the epithelial barrier and sterile tissues, which also has been shown to modulate the efficacy of anticancer drugs [38,39]. Colorectal cancer (CRC) is one of the most common cancers, its incidence ranks third and the mortality rate ranks second in the world, as well as more and more studies have shown that gut microbiota is related to the occurrence, progression and metastasis of CRC [40]. Elevated abundances of *Fusobacterium nucleatum*, *Escherichia coli*, *Bacteroides fragilis*, *Enterococcus faecalis*, *Streptococcus cholangiolyticus*, and *Peptostreptococcus* were frequently detected in the feces of CRC patients, while *Roseburia*, *Clostridium*, *Faecalibacterium*, and *Bifidobacterium* were reduced, of which *Fusobacterium* has potential as a biomarker [41]. Studies have found that *Fusobacterium* adhesin A (FadA) is also frequently detected, which can interact with E-cadherin on the endothelium and regulate the E-cadherin/β-catenin pathway to promote tumorigenesis and development [42]. Pancreatic cancer (PC), one of the highest mortality cancers, is also closely associated with dysbiosis of gut microbiota. *Helicobacter pylori*, *Fusobacterium*, and *Porphyromonas gingivalis* were significantly more abundant in PC patients, and interestingly, *Enterococcus* and *Enterobacter* were found in bile, suggesting a possible role in the transport of gut microbiota to pancreatic tissue [43]. In addition, the gut microbiota is also related to gastric cancer, breast cancer, liver cancer, prostate cancer and others, which will not be discussed in detail here [25,42,44,45,46].

### 3.3. Neurodegenerative Disease

Alzheimer’s disease (AD) and Parkinson’s disease (PD) are two common neurodegenerative diseases for which no effective treatment is available yet. With the deepening of investigation, the concept of the brain–gut axis has been further extended to the concept of the microbe–gut–brain axis, which has been confirmed in the clinic, and gut microbiota holds promise as a potential diagnostic and therapeutic target for neurodegenerative diseases, autism and depression [46]. A common feature of PD and AD patients is the presence of *Helicobacter pylori* infection. For PD studies, increased *Proteobacterial* abundance is consistent not only in clinical patients but also in animal models [47]. In AD patients, gut microbiota of a high abundance of pro-inflammatory (*Escherichia*/*Shighella*) and a low abundance of anti-inflammatory (*Escherichia rectale*) were detected, which together promoted the expression of pro-inflammatory factors [48].

### 3.4. Autism Spectrum Disorder (ASD)

ASD is a heterogeneous group of neurodevelopmental disorders, which is characterized by deficits in communication, sociality, and cognition. However, most patients had severe gastrointestinal disorders meanwhile, providing insights into the relationship between ASD and gut microbiota [49]. After a number of experimental comparisons and analyses, it can be determined that *Clostridium* spp. increased in the gut microbiota in children with autism, while *Bifidobacterium* spp. decreased, compared with healthy controls [50]. Gil Sharon et al. colonized ASD patients with gut microbiota in germ-free mice by fecal microbiota transplantation (FMT) to induce hallmark autistic behaviors, thus illustrating the possible causal link between ASD and gut microbiota [51].

### 3.5. Kidney Diseases

The kidney is an important organ for maintaining homeostasis (acid-base balance, water balance, glucose homeostasis) [52,53], and existing studies have shown that gut microbes are closely related to kidney disease and have a potential role in regulating the prognosis of kidney disease [54]. The concept of the gut–kidney axis has also been gradually extended to the brain–gut–kidney axis and the gut–kidney–mind axis, showing the close relationship between gut microbes and kidneys and other diseases [55,56,57]. FengXia Li et al. measured the intestinal bacteria of clinical patients and found that *Parasutterella*, *Rothia*, *Lactobacillus*, *Olsenella*, *Paraprevotella*, *Lactococcus*, and *Helicobacter* were highly expressed and positively correlated with the disease in patients with chronic kidney disease (CKD), while *Akkermansia*, *Lactobacillus*, *Parasutterella*, and *Clostridium IV* were negatively correlated, and the former two may be potential markers for the diagnosis of CKD [58]. It has been reported that in acute kidney injury (AKI) caused by ischemia-reperfusion, the relative abundances of *Escherichia* and *Enterobacter* were increased, while the relative abundances of *Lactobacillus*, *Ruminococcaceae*, *Faecalibacterium* and *Lachnospiraceae* were decreased [59].

**Table 1:** Gut microbiota and disease.

Disease	Gut Microbiota	Mechanism	Ref.
Obesity	*Firmicutes*, *Proteobacteria*, *Fusobacterium*, *Lactobacillus*, *Firmicutes/Bacteroidetes ratios* ↑	N/A	[31]
	*Bacteroidetes*, *Faecalibacterium palau*, *Akkermansia*, *Methanobacter smithii*, *Bifidobacterium* ↓		
Type II Diabetes	*Ruminococcus*, *Fusobacterium*, *Brucella* ↑	LPS ↑SCFA ↓	[36,37]
	*Bifidobacterium*, *Akkermansia*, *Bacteroides*, *Faecalibacterium*, *Rothella* ↓		
Colorectal Cancer	*Fusobacterium nucleatum*, *Escherichia coli*, *Bacteroides fragilis*, *Enterococcus faecalis*, *Streptococcus cholangiolyticus*, *Peptostreptococcu* ↑	Genotoxicity (DNA damage), Gut Barrier Disruption, Inflammation ↑	[41]
	*Roseburia*, *Clostridium*, *Faecalibacterium*, *Bifidobacterium* ↓		
Pancreatic Cancer	*Helicobacter pylori*, *Fusobacterium*, *Porphyromonas gingivalis* ↑	NF-κB, MAPK signaling pathways ↑	[43]
	*Enterococcus*, *Enterobacter* (in bile)		
Gastric Cancer	*Helicobacter pylori*, *Lactobacillus coleohominis*, *Klebsiella pneumoniae*, *Acinetobacter baumannii* ↑	MAP kinase, ERK1/2, VEGF, Wnt/β-catenin ↑	[42]
	Porphyromonas, Neisseria, the TM7 group, *Prevotella pallens*, and *Streptococcus sinensis* ↓		
Alzheimer’s Disease	*Helicobacter pylori*, *Escherichia*, *Shighella* ↑	Proinflammatory cytokines ↑	[48]
	*Escherichia rectale* ↓		
Autism Spectrum Disorder	*Clostridium* spp. ↑	Amino acid metabolism (Taurine)	[50,51]
	*Bifidobacterium* spp. ↓		
Chronic Kidney Disease	*Parasutterella*, *Rothia*, *Lactobacillus*, *Olsenella*, *Paraprevotella*, *Lactococcus*, *Helicobacter* ↑	IL-10, IL-4, IL-6	[58]
	*Akkermansia*, *Lactobacillus*, *Parasutterella*, *Clostridium* IV ↓		
Acute Kidney Disease	*Escherichia*, *Enterobacter* ↑	IL-17, TNF-α, IFN-γ	[59]
	*Lactobacillus*, *Ruminococcaceae*, *Faecalibacterium*, *Lachnospiraceae* ↓		

Note: Nuclear Factor Kappa B, NF-κB; Mitogen-Activated Protein Kinase, MAPK; Extracellular Reg ulated Protein Kinases, ERK; Vascular Endothelial Growth Factor, VEGF; Interleukin, IL; Tumor Necrosis Factor-α, TNF-α; Interferon-γ, IFN-γ. The meaning of a symbol in the table: ↑, increased; ↓, decreased.

## 4. Therapeutic and Sensitizing Effects of Gut Microbiota on Disease Treatment

**Figure 2:**
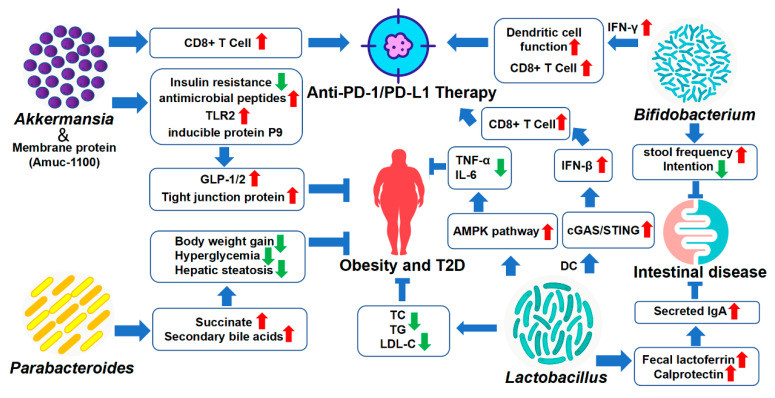
Therapeutic and sensitizing effects of gut microbiota on disease treatment. The application of gut microbiota in the treatment and prevention of diseases has been in use for a long time, and with the deepening of research, new probiotics have emerged. Except for the common *Bifidobacterium* and *Lactobacillus*, *Akkermansia* and *Parabacteroides* are expected to become a new generation of probiotics, with the potential to treat diseases such as obesity. What is more, *Akkermansia* and *Bifidobacterium* also show promise in sensitizing PD-1/ PD-L1 therapy. Note: Toll-like Receptors 2, TLR2; Glucagon-like Peptide-1, GLP-1; Programmed Cell Death 1, PD-1; Programmed Cell Death-Ligand 1, PD-L1; Total Cholesterol, TC; Triglyceride, TG; Low-Density Lipoprotein Cholesterol, LDL-C; Dendritic Cells, DC. The meaning of a symbol in the table: red arrow, up-regulated; green arrow, down-regulated.

In recent years, more and more studies have been conducted to clarify the feasibility of using probiotics from the gut microbiota to treat various diseases, or sensitizing widely-used drug efficiency. With the clinical breakthroughs of FMT, researchers are more interested in a single or several definite probiotic bacteria inoculation into the colorectum. In the following, I mainly select several hot-spot star genera for introduction, such as *Akkermansia*, *Bifidobacterium*, *Lactobacillus* and *Parabacteroides* (Figure 2).

### 4.1. Akkermansia

*Akkermansia* is a genus in the phylum *Verrucomicrobiota*, and helps to regulate the thickness of the intestinal mucosa layer and has been shown to strengthen the therapeutic outcomes of chronic disease caused by a leaky gut, inflammation, insulin resistance, and so on [60]. *Akkermansia muciniphila* (*A. muciniphila*) is the most widely studied species, a strictly anaerobic bacterium that colonizes the outer mucosa, uses mucin as the sole carbon and nitrogen source, and is considered a promising probiotic candidate [60]. In healthy people, *A. muciniphila* constitutes 3–5% of all gut microbiota and is one of the most abundant single species [61]. A large number of studies have shown that *A. muciniphila* is inversely correlated with obesity, T2D, IBD and tumors, and strategies for supplementing this bacteria to ameliorate these diseases are also emerging and some clinical trials are being undertaken [62,63,64,65].

Numerous studies have shown that animals receiving live *A. muciniphila* no longer exhibit insulin resistance and infiltration of inflammatory cells (CD11c) in adipose tissue. Live *A. muciniphila* restores endogenous production of antimicrobial peptides, and also increases endogenous production of lipids of the cannabinoid family with anti-inflammatory activities that regulate endogenous production of gut peptides involved in glucose regulation and the gut barrier, respectively, glucagon-like peptide-1 and 2 (GLP-1 and GLP-2) [66]. The current research shows that the safety of this bacteria translocation is satisfactory. In a clinical trial of broad-spectrum antibiotic therapy, two patients with *A. muciniphila* prevalence greater than 40% did not show significant signs of intestinal discomfort [67]. The first clinical assessment of the safety of live and pasteurized *A. muciniphila* in obese patients showed that oral administration for two weeks was well tolerated [62]. A recent clinical trial shows that daily oral administration of 10^10^ live or pasteurized *A. muciniphila* is safe and can improve insulin sensitivity in obese patients and reduce blood indicators related to liver dysfunction and inflammation, and it is worth mentioning that *A. muciniphila* showed a more pronounced effect [63]. Interestingly, in addition to the potential shown by live and pasteurized *A. muciniphila*, some of its membrane and secreted proteins also have beneficial effects. Membrane protein Amuc-1100 exhibits similar effects as *A. muciniphila* in improving the metabolism of obesity and diabetes in mice, and it may be that *A. muciniphila* activates Toll-like receptor 2 through Amuc-1100, regulates the expression of various tight junction proteins, and improves the intestinal tract barrier. For the induction of antimicrobial peptides, the mechanism of action of the live *A. muciniphila* and Amuc-1100 is not the same [62]. *A. muciniphila* also secretes an inducible protein P9 of GLP-1, interacting with intercellular adhesive molecules 2 (ICAM-2), promoting the secretion of GLP-1, which can improve glucose homeostasis and amelioration of metabolic disease in mice [68].

PD-1/PD-L1 immune checkpoint inhibitor (ICI) therapy is currently an important treatment method for cancer therapy, but its usage is limited due to a lower response rate. *A. muciniphila* has achieved inspiring results in sensitizing the efficacy of PD-1/PD-L1 ICI. A clinical study showed that the relative abundance of intestinal *A. muciniphila* was higher in metastatic renal cell carcinoma (mRCC) patients who responded to PD-1/PD-L1 ICI, while the lower was not responsive to it, indicating that *A. muciniphila* has the effect of sensitizing the efficacy of PD-1/PD-L1 ICI [69]. Bertrand Routy et al. found that FMT from patients who responded to PD-1/PD-L1 ICI in sterile or antibiotic-treated mice improved ICI efficacy, whereas FMT from non-responders failed to do so [70]. Pasteurized *A. muciniphila* and outer membrane protein Amuc-1100 attenuate colitis and colitis-associated colorectal cancer (CAC) by enhancing the activation and proliferation of CD8+T cells [62]. For Non-Small-Cell Lung Cancer (NSCLC) patients treated with PD-1/PD-L1 ICI, the relative abundance of intestinal *A. muciniphila* may predict prognosis, and accurate quantification of the relative abundance of intestinal *A. muciniphila* and PD-L1 expression in NSCLC patients may be the most important biomarker for outcome of immunotherapy [71].

From the above examples, we can see the potential of *A. muciniphila* as the next generation of probiotics. It not only has the potential to treat obesity and diabetes, but also has a certain effect on immunotherapy sensitization, and has well-tolerated oral safety. Surprisingly, pasteurized *A. muciniphila* seemingly has a better curative effect compared with live bacterial colonization. Some outer membrane proteins and secreted proteins of *A. muciniphila* also have certain therapeutic and sensitizing potential.

### 4.2. Bifidobacterium

*Bifidobacterium* is a Gram-positive bacteria, strictly anaerobic, non-spore-forming, capable of producing lactic acid, with a strong antibacterial effect. As a classic probiotic, *Bifidobacterium* has been widely used in the food and pharmaceutical industries and has been widely used in the supplemental treatment of constipation. *Bifidobacterium animalis* subsp. *lactis* HN019 (HN019) was well tolerated and improved stool frequency, relieving tension in patients with chronic idiopathic constipation in a 28-day clinical trial [72]. Many clinical trials have shown that *Bifidobacterium longum* alone or in combination can effectively improve the symptoms of IBD patients, and the probiotic product VSL#3 can effectively reduce rectal bleeding in IBD patients with less recurrence [73]. An in vivo study showed that oral administration of *Bifidobacterium* (*B. breve* and *B. longum*) alone can achieve almost the same effect as PD-1/PD-L1 ICI in mouse subcutaneous B16.SIY melanoma and the combination almost abolishes the tumor growth. Promoting dendritic cell function leading to enhanced CD8+ T cell priming and accumulation in the tumor microenvironment may contribute to its anti-cancer or sensitization effect [74]. Se-Hoon Lee et al. combined with clinical data found that patients who responded to PD-1 treatment had high expression of *Bifidobacterium bifidum*, and showed through abolition experiments that specific *Bifidobacterium bifidumn* strains (K57, K18 and MG731) can produce interferon-γ by to enhance T cell activation to enhance the anti-tumor effect of PD-1 therapy [75]. As a veteran of probiotics, with deep digging in various fields, supplemental *Bifidobacterium* may continue to bring more prospects as an adjuvant therapy to diverse diseases.

### 4.3. Lactobacillus

*Lactobacillus rhamnosus* (*L. rhamnosus*) is a species of the genus *Lactobacillus* and one of the most widely used probiotics. More and more studies have shown that these bacteria also have the effect of preventing obesity, anti-depression, asthma, and so on. Mo Yang et al. showed that *L. rhamnosus* JL1 administration can reduce liver injury index, TC, TG and LDL-C, which prevents obesity caused by a high-fat diet, and improves liver inflammation by activating the adenosine 5‘-monophosphate (AMP)-activated protein kinase (AMPK) pathway to reduce TNF-α and IL-6 increased by excess fat intake [76]. Yunpeng Liu et al. revealed that the anxiolytic and antidepressant effects of oral *L. rhamnosus* JB-1 are achieved through activation of CD4+CD25+T cells [77]. Pit-YeeVoo et al. showed that the combined use of *L. rhamnosus* and corticosteroids (prednisolone) to treat a mouse model of asthma showed that 50 uL of prednisolone combined with *L. rhamnosus* was more effective than 75 uL of prednisolone Solomon alone, which can reduce airway resistance and serum IgE and IgG1, inhibit the production of IL-4, IL-5, IL-6, IL-8, IL-13 and IL-17, up-regulate the production of serum IgG2a and Th1 immune responses were enhanced and further improved at the pathological level [78]. Recent studies have shown that *L. rhamnosus* exerts its antitumor activity by inducing IFN-β production through the cGAS/STING/TANK-binding kinase 1/interferon regulatory factor 7 axis in DCs and can enhance anti- PD-1 immunotherapy [79].

In addition to *L. rhamnosus*, clinical studies have shown that *Lactobacillus casei variety rhamnosus* (Lc) can not only restore the number of intestinal probiotics (*Lactobacillus* and *Bifidobacterium*), regulate the gut microbiota, but also increase levels of secreted IgA by reducing intestinal inflammatory responses (e.g., fecal lactoferrin and calprotectin) [80]. What is more, a clinical trial showed that Lactobacillus reuteri enhanced the efficacy of beclomethasone in the treatment of asthma in children and adolescents, improved the Asthma Control Test scores, and increased the peak expiratory flow [81]. It is not difficult to see that *Lactobacillus* has clear potential effects against obesity, inflammatory hepatitis, anxiety depression, asthma, and tumors and relative clinical studies are still being carried out to confirm its real beneficial effect in the future.

### 4.4. Parabacteroides

In recent years, *Parabacteroides* as a possible probiotic has gradually emerged and has been considered to be helpful in the treatment of obesity, chronic obstructive pulmonary disease, epilepsy and acute pancreatitis. *Parabacteroides* administration reduces neutrophil infiltration in acute pancreatitis (AP) by producing acetate, thereby attenuating endoglycosidase heparanase (Hpa)-induced AP [82]. Oral administration of the gut commensal *Parabacteroides goldsteinii* improves cigarette smoking (CS)-induced chronic obstructive pulmonary disease (COPD) in a mouse model with better safety, reduces intestinal inflammation and enhances cellular ribosomes and mitochondria in CS mice active [83]. *Parabacteroides distasonis* have metabolic benefits of reducing body weight gain, hyperglycemia, and hepatic steatosis in ob/ob and high-fat diet (HFD)-fed mice, and play a key role in regulating host metabolism through the production of succinate and secondary bile acids [84]. New research shows that *Parabacteroides goldsteinii* MTS01 can improve the gut microbiota composition in a mouse model of *Helicobacter pylori* infection, and reduce serum triglyceride and cholesterol levels, reducing the level of gastric inflammation(COX-2, IL-1β, and TNF-α) [85]. Although *Parabacteroides distasonis* has therapeutic effects, recent studies suggest that it may induce depression-like behavior in a mouse model of Crohn’s disease, so further in-depth research is needed for its usage [84]. A combined trial of *Akkermansia* and *Parabacteroides* showed that a combination of these two bacteria decreases gamma-glutamyltranspeptidase activity and gamma-glutamylation production and shows seizure protection in vivo [86]. *Parabacteroides* is a new type of bacteria that has only been studied in recent years and has shown beneficial effects in obesity, pancreatitis, *Helicobacter pylori* infection and other diseases. It is expected that it will become a new bacteria that can enter the clinic.

## 5. Conclusions

The gut microbiota has been one of the research hotspots in recent years, which is symbiotic with the human body and is closely related to the health and physiological functions of the human body. The gut microbiota is affected by various factors such as diet, drugs, environment, and genetics, as well as the fact that its dysbiosis is associated with many diseases. Not all gut microbiota has beneficial effects, and there are also some pathogenic bacteria, and even the beneficial/harmful effects of the same bacteria in different diseases are inconsistent and dependent on specific physiological conditions. With the continuous advancement of research and technology development, people have gradually discovered the therapeutic and sensitizing effects of certain intestinal bacteria. FMT has achieved big success in clinical practice and has become an important treatment for *Clostridium difficile* (CDI) infections that are prone to recurrence, refractory treatment and multiple complications [87]. With the deepening of research, the real value of FMT using a single genus or a combination of several genera will be thoroughly demonstrated. This review summarizes several genera of *Akkermansia*, *Bifidobacterium*, *Lactobacillus* and *Parabacteroides* that have therapeutic and sensitizing potential. It is hoped that further research will speed up the application of these probiotics in human health.

## Data Availability

Not applicable.
